# Determinates of muscle precursor cell therapy efficacy in a nonhuman primate model of intrinsic urinary sphincter deficiency

**DOI:** 10.1186/s13287-016-0461-6

**Published:** 2017-01-06

**Authors:** James Koudy Williams, Ashley Dean, Shannon Lankford, Tracy Criswell, Gopal Badlani, Karl-Erik Andersson

**Affiliations:** 1 Wake Forest Institute for Regenerative Medicine, Wake Forest Baptist Medical Center, Winston-Salem, NC USA; 2 Department of Urology, Wake Forest University Baptist Medical Center, Winston-Salem, NC 27157 USA; 3 Department of Clinical Medicine, Aarhus University, Aarhus, Denmark; 4 Wake Forest Institute for Regenerative Medicine, Wake Forest University, 391 Technology Way, Winston-Salem, North Carolina 27101 USA

**Keywords:** Nonhuman primate, Cell therapy, Aging, Obesity, Dysmenorrhea, Urinary incontinence

## Abstract

**Background:**

Cell therapy for intrinsic urinary sphincter deficiency (ISD) in women has been moderately effective, and improvements are needed. To improve treatment efficacy, it is important to better understand determinates of cell efficacy in the different patient cohorts. We have reported that in nonhuman primates the chronicity of ISD may affect cell efficacy, but additional factors (age, psychosocial stress, hormone status, body weight) can be associated with many disease/treatment outcomes in women – and these factors are the focus of this study.

**Methods:**

Adult female cynomolgus monkeys were divided into groups: (1) younger (*n* = 10, 5–8 years of age) versus older (*n* = 10, 13–18 years of age); (2) age-matched/socially subordinate (*n* = 15) versus socially dominant (*n* = 15); and (3) age-matched lower body weight (*n* = 6) versus higher body weight (*n* = 6). Autologous skeletal muscle precursor cells (skMPCs, 5 million) were injected into the urinary sphincter 6 weeks after a surgically induced ISD procedure. Resting and pudendal nerve-stimulated maximal urethral pressures (MUP) were measured before, and 3 and 6 months post-skMPC treatment and urinary sphincter muscle/collagen content within the sphincter complex was measured by quantitative histology 6 months posttreatment.

**Results:**

Efficacy of skMPCs on MUP and sphincter muscle/collagen ratios are affected by age (average 40% reduction in efficacy, *p* < 0.05 vs. younger NHPs), social stress (average 30% reduction in efficacy, *p* < 0.05 vs. socially dominant) and body weight/fasting glucose concentrations (average 35% reduction in efficacy, *p* < 0.05 vs. lower body weight).

**Conclusion:**

Multiple factors (age, stress-induced dysmenorrhea, and body weight) affect the efficacy of cell therapy to restore structure and function in the urinary sphincter complex in NHPs with ISD. Consideration of, and alternatives for, these patient cohorts should be considered.

## Background

Intrinsic urinary sphincter deficiency (ISD) is a common cause of stress urinary incontinence (SUI) and remains a significant quality-of-life issue. It is a chronic condition resulting from aging and childbirth injury to the urinary sphincter musculature and innervation, becoming clinically evident in the peri/postmenopausal years [1, 2]. Although good results of surgical therapy of SUI have been reported [3], complications are not infrequent [4] and alternative treatments may be desirable, particularly when surgical treatment has failed or if surgery poses too great a risk. Studies using adult skeletal muscle precursor cells (skMPCs) to induce tissue regeneration and repair of the damaged urethral sphincter have shown positive results both in animals [5, 6] and humans [7]. However, cell therapy for ISD in women has been only moderately successful with consistent 50% beneficial effects in around 50% of women. In contrast, results of preclinical cell studies have been more optimistic [6]. This apparent difference in efficacy could be due to multiple issues with animal and disease modeling, as preclinical studies are usually performed on adult, healthy animal models. Clinical studies, even with strict inclusion/exclusion criteria, test treatments in a much more diverse population.

Factors known to affect the efficacy of cell therapy are chronicity of disease, age, hormonal status, and body weight. We recently reported reduced efficacy of skMPCs in nonhuman primates (NHPs) with chronic (versus acute) intrinsic urinary sphincter deficiency (ISD) [8]. Senescence of stem cells, associated with aging is reported to dramatically reduce the effects of muscle regeneration and the efficacy of muscle cell therapy [9]. Pelvic health in women has also been shown to be influenced by estrogen deficiencies [10, 11], which occur as early as perimenopause (35–50 years of age) and in physically or mentally stressed women. Body weight and prediabetic conditions can dramatically increase the risk of diseases such as urinary incontinence, and the effects of cell therapy in this population are unclear [12].

For the past 8 years, we have examined the effects of autologous skMPC therapy for ISD in female NHPs. Results consistently showed improvement in urinary sphincter structure and function. However, there was variability in the data. The goal of the present study was to re-examine the data in the context of cell efficacy in older versus younger; heavier versus lighter, and dominant versus subordinate NHPs.

## Methods

### Animal model

Studies in adult female nonhuman primates (*Macaca fascicularis*) were approved by the Wake Forest University Institutional Animal Care and Use Committee protocol number A16-012 and were performed in compliance with the Animal Welfare Act and the Guide for the Care and Use of Laboratory Animals. Euthanasia was performed according to the standards of the American Veterinary Medical Association.

### Design

Over the past 8 years we have studied the effects of autologous skMPC therapy on acute ISD in over 50 female cynomolgus monkeys. *Aging analysis*: the ages of the animals ranged from 5 to 18 years. A sexually mature female monkey is approximately 4 years of age and lives for approximately 25 years [13, 14]. Monkeys have a 28-day menstrual cycle and go through menopause between 22 and 25 years [13, 14].

We retrospectively analyzed the effects of skMPC therapy in the ten oldest (13–18 years - equivalent to older cycling women) and the ten youngest (5–8 years - equivalent to younger cycling women) monkeys from the total number of monkeys studied. *Body weight analysis*: the monkeys varied in body weight from 2.8 kg to 6.7 kg. We compared skMPC treatment effects in six age-matched (10 years of age) heavier NHPs (5.0–6.7 kg) versus lighter NHPs (2.8–3.4 kg body weight). *Social status analysis*: female monkeys, when housed in social conditions, naturally sort themselves into dominant versus subordinate social status [13, 14]. Subordinate female NHPs of this species develop impaired menstrual cyclicity, relative hypoestrogenism, increased production of stress cortisol, and psychosocial symptoms of stress (passive behavior toward the dominate NHPs). For this study, we selected 15 dominant and 15 subordinate NHPs from the total of 50 and compared the efficacy of skMPCs on ISD.

### The NHP model of ISD

The ISD procedure was done after baseline urodynamic measures were obtained. The monkeys were sedated with ketamine 10–15 mg/kg IM, and 1–5% isoflurane was used for induction and maintenance of anesthesia. Monkeys were prepared for aseptic surgery, anesthetized, and a lower midline abdominal incision (4 cm in length) made to expose the pelvic area of the abdomen. The distal urinary tract was approached using gentle dissection of connective tissue just ventral to the urinary bladder extending dorsally to the bladder neck and caudally 2 cm to either side of the rhabdosphincter. The pudendal nerve branches supplying the sphincter (usually three in number) were identified and then selectively electrocauterized - while not damaging the sphincter directly - and then transected [15, 16]. Special care was taken not to damage surrounding structures. The abdomen was closed in two layers and postoperative support given.

### skMPC isolation and injection

A 1 cm^3^ sample of quadriceps muscle was aseptically removed from anesthetized NHPs and transported in a wash solution of Dulbecco’s phosphate-buffered saline (DPBS, HyClone, South Logan, UT, USA) with 1% antibiotic/antimitotic (HyClone). The tissue was washed 10 minutes × 3 in fresh wash solution with periodic gentle agitation with a final rinse in DBPS. The sample was trimmed of unwanted tissue, weighed, and minced into fragments approximately 0.5 mm^2^ or less. Digestion media consisting of 2:1 dispase II (Sigma-Aldrich, St. Louis, MO, USA): collagenase type I (Worthington, Lakewood, NJ, USA) per ml of basal media (custom-designed muscle progenitor cell media, PeproTech, Rocky Hill, NJ, USA) was added to the minced tissue at 1 ml per 100 mg of tissue. The sample was incubated at 37 °C, 5% CO_2_ for 45 minutes. Upon completion, the digestion was terminated using 2× volume of growth media (PeproTech basal media plus FBS and custom growth supplements) to digestion media and rigorous pipetting was applied. The suspension was filtered through a 100-micron filter and centrifuged for 5 minutes at 1500 RPM. The supernatant was aspirated, fresh growth media was added, and spun for a second time. Then the sample was plated on a pretreated collagen I 100-mm culture plate (BD Biocoat, Becton Dickinson, Franklin Lakes, NJ, USA) and incubated for 24 hours at 37 °C, 5% CO_2_. The following day, the aspirate was collected and replated on a new pretreated collagen-coated plate to reduce fibroblast contamination in the cell culture. The skMPCs were isolated and characterized as described previously [16]. Eight weeks following collection of the sample, 5 million skMPCs were suspended in 2 milliliters of DMEM without serum and injected directly into the urinary sphincter complex [at the level of the sphincter skeletal muscle layer and at four locations (12, 3, 6, and 9 o’clock positions)] of anesthetized monkeys as described previously [15, 16].

### FACS analysis

Cell cultures from passage 2 skMPCs were analyzed by fluorescence-activated cell-sorting analysis (FACS analysis), 200,000 cells of each of the fixed samples from the monkeys were immunolabeled. All cell samples were pelleted and blocked with the use of Fc Blocking Reagent (Miltenyi Biotec Inc., Auburn, CA, USA) for 15 minutes. Immunolabeling followed immediately with mouse monoclonal anti-CD34 (550619, BD Pharmingen, San Diego, CA, USA) antibody, mouse monoclonal anti-CD44 (550989, BD Pharmingen) antibody, mouse monoclonal anti-CD45 (558411, BD Pharmingen), skeletal muscle actin (1:100, Thermo Fisher Scientific, Waltham, MA, USA, PA1-37019), MyoD (myoblast determination protein) (1:100, Pharmingen), or CD117 (1:100, BioLegend, San Diego, CA, USA, 104D2). After incubating for 1 hour at 4 °C all tubes were washed three times and labeled using species-specific FITC (FI-1000, Vector Laboratories, Burlingame, CA, USA). A negative control (no primary antibody, species-matched serum) was run simultaneously with each experimental sample. Cell fluorescence was measured immediately after staining with a Becton Dickinson FACS Calibur flow cytometer (BD Biosciences, San Diego, CA, USA) and data analyzed using FlowJo software v. 7.1.3 (Tree Star Inc., Ashland, OR, USA).

### Sphincter function

Monkeys were sedated and anesthetized and urodynamic measures recorded at baseline (before the nerve injury), and then prior to injections, at 3 months, and 6 months post-injection using the Life-Tech Urolab Opus System V (Life-Tech Inc., Stafford, TX, USA) in combination with a 6 French Millar microtip transducer catheter (Millar Instruments Inc., Houston, TX, USA) and a rectal balloon catheter. The catheter was inserted transurethrally and the residual urine evacuated. Urethral pressure profilometry was performed by automatic withdrawing of the sensor catheter at 0.5 mm/s. Rectal pressure was measured with a balloon catheter attached to a transducer (Life-Tech Inc.). Using the pressure sensors at its tip (direction upward), a static urethral pressure profile was recorded on the urodynamic machine and the maximal urethral pressure (MUP) in the region of external sphincter recorded [15, 16]. This process was repeated three times and the mean of the urethral pressure measurements calculated for each animal. MUP was then measured during pudendal nerve stimulation.

### Collection and analysis of tissues

The monkeys were euthanized for tissue retrieval using sodium pentobarbital (80–100 mg/kg/intravenously). The urethra, approximately 1 cm in length, was removed from the animal, and immersion fixed in 2% phosphate-buffered formalin for 48 hours and then transferred to 70% ethanol. Tissue sections were dehydrated in increasing concentrations of ethanol, embedded in paraffin, and cut into 5-μm-thick cross sections. Sections used to quantify sphincter collagen and muscle content, spaced evenly along the length of the urethra, were fixed and stained with Masson’s Trichrome, which stains collagen fibers blue and muscle fibers red. Image analysis was done using the Image-Pro AMS 6.0 software (Media Cybernetics, Bethesda, MD, USA).

### Statistical analysis

MUP and collagen/muscle data were first analyzed with a one-way ANOVA to detect differences among groups. Logarithmic transformation was used if data were not distributed normally around the mean. If the ANOVA was significant, post hoc analysis (between groups) was performed using unpaired Student’s *t* test with a Holm-Sidak correction for multiple groups. *p* < 0.05 was considered statistically significant. Data are presented as mean plus/minus standard error of the mean.

## Results

### Cells

**Fig. 1:**
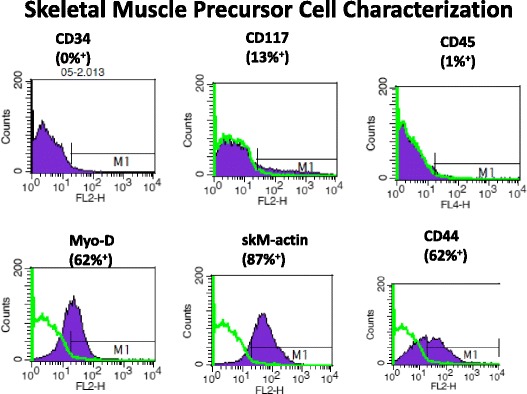
FACS analysis of skMPCs. Fluorescence-activated cell-sorting analysis (FACS analysis) was performed on passage 2 skMPCs from the monkeys included in this retrospective analysis. This figure represents the findings from one monkey in the ISD/skMPC-treated group of a younger (5-year-old), normal weight (3.1 kg), dominant monkey. The percentage of FITC-labeled cells (compared to serum controls) is shown for each of the cell markers

Passage 2 cells were analyzed using fluorescence-activated cell sorting (FACS). The isolated skMPCs expressed markers for the muscle precursor marker Myo-D, the stromal marker CD44 and skeletal muscle actin (Fig. 1). There was very little expression for the early precursor cell markers CD177, CD34, or Oct4 (Fig. 1). Interestingly, there was very little difference in expression patterns between young and old; heavier versus lighter; or dominant versus subordinate cell cultures. While not shown, there was also very little difference in the doubling time among the different cell cultures.

### Effects of age

**Fig. 2:**
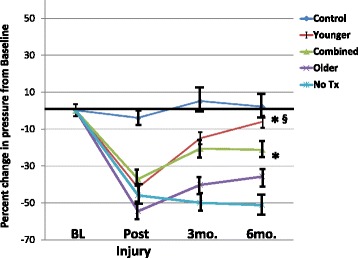
Maximal urethral pressures (MUP) in the older versus younger monkeys. This figure depicts the percent changes in maximal urethral pressure (MUP) from baseline values (before injury) in: the “Control” group (no ISD/no treatment); ISD/no treatment “No Tx” group; the “Combined” (ISD/skMPC treatment group); the “Younger” (age 5–8 years) and the “Older” (age 13–15 years) groups. ^*^ *p* < 0.05 vs. NoTx. ^§^ *p* < 0.05 vs. older. Values are mean ± SEM

**Fig. 3:**
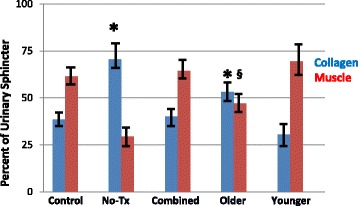
Sphincter muscle/collagen content in the older versus younger monkeys. This figure depicts the percent of urinary sphincter complex that is muscle versus collagen as determined from cross-sectional areas of the sphincter stained with Mason’s Trichrome. These values are shown for: the “Control” group (no ISD/no treatment); ISD/no treatment “No Tx” group; the “Combined” (ISD/skMPC treatment group); the “Younger” (age 5–8 years) and the “Older” (age 13–15 years) groups. ^*^ *p* < 0.05 vs. Control. ^§^ *p* < 0.05 vs. Younger. Values are mean ± SEM. The statistical significance markers reflect changes in both the collagen and muscle content

Figures 2 and 3 depict the effects of skMPC therapy on maximal urinary sphincter pressure (MUP), and sphincter collagen/muscle content in younger (5–8 years of age) versus older (13–18 years of age) monkeys. MUP values (Fig. 2) in the combined and younger animals were greater than the ISD/no treatment groups (*p* < 0.05). MUP values in younger animals was greater than that of older animals (*p* < 0.05). The effects of age on sphincter collagen/muscle content (Fig. 3) mirror that of the MUP results. The injury procedure changed the collagen/muscle ratio from a muscle-dominant to a collagen-dominant sphincter (*p* < 0.05). Treatment with skMPC increased the amount of muscle in the urinary sphincter in the combined and the younger groups (*p* > 0.05 vs. Control), but not the older group (*p* < 0.05 vs. Control and younger group). There were not enough numbers of NHPs to do an interactive assessment of age and body weight with age and social status.

### Body weight

**Fig. 4:**
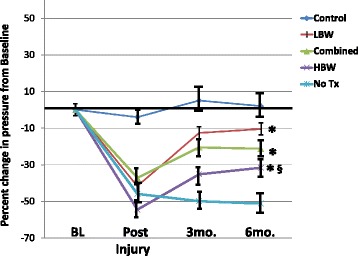
Maximal urethral pressures in the heavier versus lighter monkeys. This figure depicts the percent changes in maximal urethral pressure (MUP) from baseline values (before injury) in: the “Control” group (no ISD/no treatment); ISD/no treatment “No Tx” group; the “Combined” (ISD/skMPC treatment group); the lighter – “LBW” (2.8–3.4 kg) and the heavier – “HBW” (5.0–6.7 kg) groups. ^*^ *p* < 0.05 vs. NoTx. ^§^ *p* < 0.05 vs. LBW. Values are mean ± SEM

**Fig. 5:**
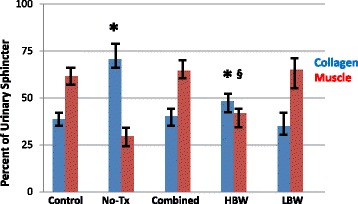
Sphincter muscle/collagen content in the older versus younger monkeys. This figure depicts the percent of urinary sphincter complex that is muscle versus collagen as determined from cross-sectional areas of the sphincter stained with Mason’s Trichrome. These values are shown for: the “Control” group (no ISD/no treatment); ISD/no treatment “No Tx” group; the “Combined” (ISD/skMPC treatment group); the lighter – “LBW” and the heavier – “HBW” groups. ^*^ *p* < 0.05 vs. Control. ^§^ *p* < 0.05 vs. LBW. Values are mean ± SEM. The statistical significance markers reflect changes in both the collagen and muscle content

Monkeys in our studies varied a great deal in their body weight. We compared the effects of skMPC in monkeys with a body weight (2.8–3.4 kg) versus those whose body weight was 5.0–6.7 kg (Figs. 4 and 5). skMPC increased the MUP values in all three groups (Combined, lower body weight - LBW, higher body weight - HBW) compared to no treatment (*p* < 0.05) (Fig. 4). However, the MUP values of the HBW group remained significantly lower than the baseline and Control groups (*p* < 0.05). Again the sphincter collagen/muscle content mirrored the effects of skMPCs on MUP values (Fig. 5). Injury produced a collagen-rich sphincter and treatment reversed this relationship to a muscle-rich lesion in the Combined and LBW groups (*p* < 0.05), but not the HBW monkeys (*p* > 0.05 vs. Injury). There were not enough numbers of animals to look at interactive effects of body weight and age or social status.

### Social status

**Fig. 6:**
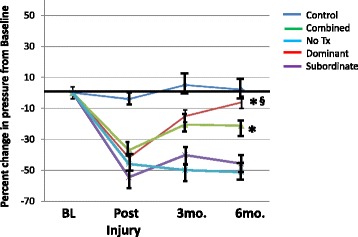
Maximal urethral pressures in the dominant versus. subordinate monkeys. This figure depicts the percent changes in maximal urethral pressure (MUP) from baseline values (before injury) in: the “Control” group (no ISD/no treatment); ISD/no treatment “No Tx” group; the “Combined” (ISD/skMPC treatment group); the “Dominant” and the “Subordinate” groups. *p < 0.05 vs. NoTx. ^§^ *p* < 0.05 vs. Subordinate. Values are mean ± SEM

**Fig. 7:**
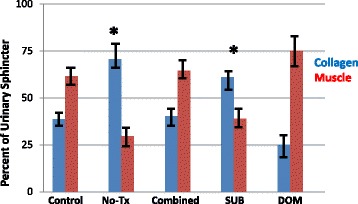
Sphincter muscle/collagen content in dominant versus subordinate monkeys. This figure depicts the percent of urinary sphincter complex that is muscle versus collagen as determined from cross-sectional areas of the sphincter stained with Mason’s Trichrome. Results are shown for the “Control” group (no ISD/no treatment); ISD/no treatment “No Tx” group; the “Combined” (ISD/skMPC treatment group); the “Dominant” and the “Subordinate” groups. ^*^ *p* < 0.05 vs. No Tx. Values are mean ± SEM

In monkeys who are pair-housed, one is the dominant and one is the subordinate animal. This creates a social stress condition where the subordinate individual has increase plasma levels of cortisol and impaired menstrual cyclicity [13, 14]. We compared the effects of skMPC therapy in these two social groups (Figs. 6 and 7). skMPC therapy in dominant monkeys had a marked effect on increasing MUP values from post-injury values (*p* < 0.05) and was the only group to restore MUP values to that of Control animals (>0.05 vs. Control) (Fig. 6). skMPC therapy improved MUP values in the combined group (*p* < 0.05 vs. no treatment), but did not restore MUP values to Control values. There was little effect of skMPC therapy on MUP in the subordinate monkeys (*p* > 0.05 vs. no treatment and *p* < 0.05 vs. Control (Fig. 6). Again, injury created a collagen-rich sphincter complex (Fig. 7). skMPC therapy restored the muscle content in the dominant and Combined groups (*p* < 0.05 vs. Injury only and *p* > 0.05 vs. Control. skMPC therapy had little effect of sphincter collagen and muscle content in the subordinate monkeys (*p* < 0.05 vs. Combined and dominant) and *p* > 0.05 vs. injury.

## Discussion

This was a subgroup analysis of previously performed experiments where the efficacy of skMPC therapy was compared between older versus younger; heavier versus lighter; and dominant versus subordinate monkeys. The rationale for these subgroup analyses was that these factors (age, body weight and social stress have been shown to influence the risk of diseases and been shown to influence, directly or indirectly the efficacy of cell therapies [8–12]. The major finding of this study was that the efficacy of skMPC therapy was reduced in older monkeys, in heavier monkeys and in monkeys with psychosocial stress – associated with reduced endogenous estrogen production.

### Aging and regeneration

Aging is probably one of the most significant risk factors for urinary incontinence in women [2, 17]. There are several lines of evidence that aging reduces the ability of cells to regenerate. Some have reported that the cells from older versus younger individuals have different regenerative capacity [9, 18, 19]. It has also been reported that muscle from older mice contain fewer regenerative cells [19]. Liu et al [18] report that the muscle environment of older individuals is not conducive to cell regeneration because of transcriptional upregulation of immune activation and downregulation of mitochondrial biogenesis. In the present study, there was no apparent difference in expression of proteins associated with muscle health, but the age range between the younger and older monkeys may not have been great enough to produce these changes and we did not examine transcription differences between the younger and older cells. Nonetheless, this age difference in the monkeys was sufficient to have effects on the efficacy of cell therapy to restore sphincter function and structure. To our knowledge, the finding that aging affects the ability of skMPCs to treat intrinsic sphincter deficiency is new. However, the mechanisms responsible for these differences have not been established. Preclinical models used to test treatment efficacy for urinary incontinence need to consider the age of the animals being used.

### Body weight and regeneration

Like aging, obesity is an important risk factor for urinary incontinence in women [20]. This association is most likely explained by metabolic changes within the muscle tissue [21] that reduce the ability of muscle cells to regenerate. Not much is known about the effects of obesity on the efficacy of cell therapy to regenerate tissues. In the present study, we compared skMPC efficacy in two weight groups of monkeys. The first averaged around 3 kg and the second around 5.5 kg. This may not seem like a big difference, but the normal weight for this species is between 2.5 and 3.0 kg and a 5.5 kg monkey is noticeably heavier with large amounts of abdominal body fat. Blood tests did not reveal any differences in fasting glucose or insulin concentrations between these two groups and no glucose tolerance tests were performed. However, there was a markedly reduced effect of skMPCs to restore urinary sphincter structure and function in the heavier monkeys despite no measurable differences in the skMPCs themselves. Again this study is limited by the lack of a definitive mechanism explaining the differences in efficacy, but emphasizes the importance of considering body weight as an important variable in cell efficacy in both preclinical and clinical studies.

### Social status/stress/estrogen and regeneration

Urinary incontinence in women is associated with changing hormonal status during the perimenopausal and postmenopausal years [2, 19]. However, it is unclear if this increased risk is due to the changes in hormones themselves, or advancing age – which is strongly linked to muscle atrophy and degeneration [18, 19]. Cynomolgus female monkeys, like women, have a 28-day menstrual cycle, natural menarche and menopause, and are social animals [13, 14]. When placed in social groups, they sort themselves into dominant and subordinate individuals. The subordinate individuals have irregular and incomplete menstrual cycles, increased production of stress hormones, and are more prone to developing vascular disease [13, 14, 22, 23]. When the female cynomolgus monkeys in this experiment were assessed for their social status, the subordinate females had a markedly reduced response to skMPC therapy compared to the dominant females. We did not measure their sex or stress hormone concentrations, but all previous studies have confirmed reduced estrogen production and elevated cortisol responses [13, 14]. The important takeaway message is that, like monkeys, people live in varying stressful conditions and women can develop relative dysmenorrhea from intense exercise and stress [13, 14, 22, 23]. While we have not compared intact versus ovariectomized monkeys (e.g., almost complete estrogen deficiency), it is important that the relative estrogen deficiency can reduce the efficacy of skMPC therapy.

### Limitations of this study

Some limitations have been mentioned. This was a retrospective study whose original purpose was not to assess subgroups. It would have strengthened the study if we had been able to define mechanisms for the physiological and structural endpoints or to examine these determinates separately and interactively. For instance, with the smaller numbers, it was not possible to determine if age and weight or age and social status interact to produce even less responsiveness to cell therapy.

## Conclusions

Age, stress-induced dysmenorrhea, and body weight may affect the efficacy of cell therapies. These findings are important in helping explain some of the differences in preclinical and clinical studies. More importantly, if valid in humans, they identify factors that may affect efficacy and optimization of cell treatment in these subgroups of patients.

## Abbreviations

HBW: higher body weight
ISD: intrinsic urinary sphincter deficiency
LBW: lower body weight
MUP: maximal urethral pressure
NHP: nonhuman primate
skMPC: skeletal muscle precursor cells
SUI: stress urinary incontinence
